# Suicidal Crises in Adolescents Treated in Adult Psychiatric Units: Clinical Features and Care Challenges

**DOI:** 10.1192/j.eurpsy.2026.12069

**Published:** 2026-07-31

**Authors:** I. Mosbah, H. Ghabi, A. Karmous, H. Nefzi, M. Karoui, F. Ellouze

**Affiliations:** 1 Department of Psychiatry G, Razi hospital, Manouba; 2 El Manar University, Faculty of Medicine of Tunis, Tunis; 3 Department of Psychiatry A, Razi hospital, Manouba, Tunisia

## Abstract

**Introduction:**

Suicide is a leading cause of death among adolescents, with suicidal crises often requiring hospitalization. In the absence of dedicated adolescent psychiatric services, 16 to 19-year-olds adolescents are frequently admitted to adult psychiatry. This setting, while necessary, may not fully address their developmental, social, and family-specific needs.

**Objectives:**

This study aimed to describe the characteristics of adolescents hospitalized for suicidal behavior, identify associated risk factors, and discuss implications for targeted prevention and adapted care strategies

**Methods:**

We conducted a retrospective descriptive study of adolescents aged 16–19 years hospitalized in an adult psychiatric department between 2019 and 2025. Sociodemographic, familial, clinical and data were collected from medical records, with a focus on suicidal behavior.

**Results:**

Between 2019 and 2025, 49 adolescents were hospitalized in the adult psychiatry unit, of whom 18 were admitted for suicidal behavior, including suicidal ideation and attempts. The mean age was 17.4 years. Females predominated (77%). Most were not attending secondary school (72%), while 16.6% were employed. More than half (55%) lived with both biological parents, one third with a single parent due to divorce or parental loss, and one case involved adoption. Psychoactive substance use was reported in one third, mainly tobacco (33.3%) and cannabis (33.3%), with one case of LSD and ecstasy use. A family history of psychiatric disorders was present in 20%, with no history of suicide.

Previous psychiatric follow-up was documented in 53.3%. Traumatic events were reported in 26.7% (neglect, divorce, parental loss). Past suicidal behavior occurred in 60% (4 cases medication, 4 phlebotomy). The current hospitalization was triggered by suicidal ideation in 46.7% and attempts in 53.3%, mostly via medication overdose (33.3%), hanging (13.3%), and self-injury (6.7%). Hospitalization was initiated by a third party in 80%, and by court order in 20%. Mean length of stay was 8.7 days.

Psychiatric diagnoses included borderline personality traits (27.7%), adjustment disorder (27.7%), and depressive episodes (16.6%). Schizophrenia and intellectual disability were each observed once, and one case of type I bipolar disorder was identified. Treatment was primarily pharmacological: antipsychotics (66.7%), antidepressants (46.7%), and mood stabilizers (20%). Structured psychotherapy was documented in over half.

**Conclusions:**

Adolescents admitted to adult psychiatry for suicidality represent a clinically complex and high-risk group, often with prior psychiatric history and significant psychosocial adversity. These findings underscore the need for tailored prevention strategies, improved continuity of care, and development of specialized adolescent pathways even within adult psychiatric services.

**Disclosure of Interest:**

None Declared

